# Temperature-dependent gas transport performance of vertically aligned carbon nanotube/parylene composite membranes

**DOI:** 10.1186/1556-276X-9-448

**Published:** 2014-08-28

**Authors:** Lei Zhang, Junhe Yang, Xianying Wang, Bin Zhao, Guangping Zheng

**Affiliations:** 1 School of Materials Science and Engineering, University of Shanghai for Science and Technology, Shanghai 200093, China; 2 Department of Mechanical Engineering, The Hong Kong Polytechnic University, Hung Hom, Kowloon, Hong Kong, China

**Keywords:** Carbon nanotube, Composite membrane, Gas transport, Parylene

## Abstract

A novel composite membrane consisting of vertically aligned carbon nanotubes (CNTs) and parylene was successfully fabricated. Seamless filling of the spaces in CNT forests with parylene was achieved by a low-pressure chemical vapor deposition (CVD) technique and followed with the Ar/O_2_ plasma etching to expose CNT tips. Transport properties of various gases through the CNT/parylene membranes were explored. And gas permeances were independent on feed pressure in accordance with the Knudsen model, but the permeance values were over 60 times higher than that predicted by the Knudsen diffusion kinetics, which was attributed to specular momentum reflection inside smooth CNT pores. Gas permeances and enhancement factors over the Knudsen model firstly increased and then decreased with rising temperature, which confirmed the existence of non-Knudsen transport. And surface adsorption diffusion could affect the gas permeance at relatively low temperature. The gas permeance of the CNT/parylene composite membrane could be improved by optimizing operating temperature.

## Background

Because of their excellent mechanical, electrical, and thermal properties, carbon nanotubes (CNTs) have been used in many areas such as conductive or electromagnetic devices, sensors, high-strength composites, and multifunctional membranes [1-4]. Single-walled carbon nanotubes (SWCNTs) or multi-walled carbon nanotubes (MWCNTs) which have atomically smooth inner surfaces could provide us with ideal systems for the investigation on the characteristics of molecular transport in the nanometer scales [5,6]. Recently, the phenomena of gas transport through CNTs embedded in polymer matrix are of great interest. The low-cost CNT/polymer composites are promising membranes which possess high transparency and extraordinary gas permeance performance [7,8].

CNT-based membranes have opened up a new prospect for the selective separation of gases [9,10]. CNTs exhibit routes of fast interfacial slip for gas molecules on their inner walls since they have a large non-interacting van der Waals distance and atomically smooth surfaces that do not scatter gas molecules. In addition, CNTs may provide uniform pore structures at the nanometer scales that can be finely tailored by controlling the catalyst particle sizes. Polymer matrix membranes with CNTs as fillers have attracted great attention since they are resilient, easily fabricated, and chemically stable. Unfortunately, random aggregations and dispersions of CNTs in the polymer matrix are usually found in the CNT/polymer membranes fabricated by a conventional solution method, which deteriorate the gas permeance performances of the membranes [11,12]. In order to synthesize high-performance membranes, a lot of efforts have been devoted to improve CNT alignments with the assistances of electrical fields, flowing gases, and surface-lattice-guided growth of CNTs or CNT sheets [13,14]. However, it remains a challenge to fabricate composite membranes in which good CNT alignment and high porosity were achieved simultaneously for high gas permeance. To address these issues, a fabrication method was developed by infiltrating vertically aligned CNTs (VACNTs) with poly-para-xylylene (parylene-C) through a vapor deposition technique [15-18]. In this work, the poly-para-xylylene (parylene-C) was chosen as matrix material to fill the intertube gaps in VACNTs because of its mechanical robustness, chemical inertness, and low permeability to moisture and gases, which are desirable for membrane applications. Furthermore, it is easy to be vapor-deposited at room temperature while providing excellent gap filling between high aspect ratio nanostructures, as will be ideal for infiltrating CNTs without sacrificing their alignment. So far, CNT forests embedded in parylene have been reported for several applications such as electrochemical sensors [15] and porous membranes [18], but it is still necessary to fully explore usage of this polymer in composite membranes for gas separation.

In the previous studies on the non-Knudsen transport phenomena in CNT-based membranes [19,20], the effects of temperature on the permeation behaviors have not been well elucidated. Therefore, we investigate the effects of temperature on the permeation behaviors of membranes containing VACNT [21]. For most gases, the permeance firstly increased as the temperature rose up to 50°C and then decreased with further increasing temperature. The changed permeance with temperature and the temperature-dependent gas permeance both suggested that the gas diffusion in CNT channels does not fully conform to the Knudsen diffusion kinetics, and other diffusion mechanisms of gas molecules might exist.

## Methods

Water-assisted chemical vapor deposition (CVD) technique was employed to synthesize VACNTs at 815°C using high-purity ethylene (99.9%) as carbon source. Al_2_O_3_ (approximately 40 nm)/Fe (1.4 nm) bilayer films were evaporated on Si (100) substrate as catalysts. Mixture of pure argon (99.999%) and H_2_ (99.999%) with a total flow rate of 600 sccm was used as the carrier gas. Water vapor was employed as catalyst preserver and enhancer and was supplied by passing a portion of the carrier gas Ar through a water bubbler [22,23]. Typically, the growth of CNT forests was carried out with ethylene (100 sccm) under a water concentration of 100 to 200 ppm for 10 s [24]. And CNT forests of 8 to 10 μm in height were obtained.

To fabricate VACNT/parylene membranes, parylene was used to impregnate the spaces among VACNTs through a low-pressure CVD method. The as-synthesized VACNTs on Si substrates were placed in a deposition instrument (Parylene Coating System-2060 V, Shanghai PAL Chetech Co. Ltd, Shanghai, P.R. China). In a vacuum of 0.1 Torr, para-xylene monomer was polymerized to form parylene films on the CNT arrays, which was kept at room temperature. Ten-micrometer-thick parylene films were deposited, and the deposition rate was kept at 1.2 μm/h. After parylene deposition, the composite membranes were heated up and held at 375°C for 1 h in Ar atmosphere to allow the parylene to reflow. Subsequently, a planar surface of the membrane was formed. The membrane was then cooled at room temperature at a cooling rate of 1°C min^-1^.

After polymer infiltration and annealing, an Ar/O_2_ plasma etching process was carried out to remove the excessive parylene and open up the CNT tips [25-27]. The Ar/O_2_ plasma oxidation processing of the samples was accomplished using a homemade plasma-enhanced CVD (PECVD) apparatus. The operating power was 100 W, and the typical etching time was 90 min. Plasma treatment on the composite membrane was performed at 100 Pa at room temperature. A 13.56-MHz RF power supply (CESAR 136, Advanced Energy Industries, Inc., CO, USA) was used to generate plasma. Ar (99.999%) and O_2_ (99.999%) were employed as feed gases, and the background vacuum of the equipment was 1 × 10^-4^ Pa. The composite membrane with opened CNT channels was then immersed in a 50% hydrogen fluoride acid solution for 24 h to remove the CNT/parylene membrane from the silicon substrate. The freestanding composite membrane [28] was washed with deionized water, followed by drying. The bottom or untreated surface of the membrane was also treated shortly by plasma etching to expose CNTs. Finally, a through-hole membrane was obtained.

It is important to exclude the gas leakage within the polymer matrix when the gas permeances through the CNTs in the composite membranes are measured. The gas leakage in the CNT/parylene composite membrane was characterized through H_2_ permeation measurement before it was treated by plasma etching. The freestanding CNT/parylene composite membrane was first sealed between two pieces of aluminum adhesive tapes with pre-punched holes (3 mm in diameter). Then, the membrane was mounted in the gas line of a permeation testing apparatus, which was purged with the target gas for several times to avoid any possible impurities. Finally, pure H_2_, He, N_2_, Ar, O_2,_ and CO_2_ (99.999%) were introduced to the upstream side of the membrane [29] for permeation measurements. A pressure or flow controller (MKS 250E, MKS Instruments, MA, USA) was connected to the upstream and downstream sides of the composite membrane to control the relative gas pressures by automatically tuning the gas feeding rates. The permeabilities at a variety of pressures (10 to 80 Torr) were measured using a mass flow meter connected at the downstream side. The measurements were carried out at different temperatures. The pore density and porosity of the membranes were measured using KCl diffusion through the membrane [30].

## Results and discussion

**Figure 1:**
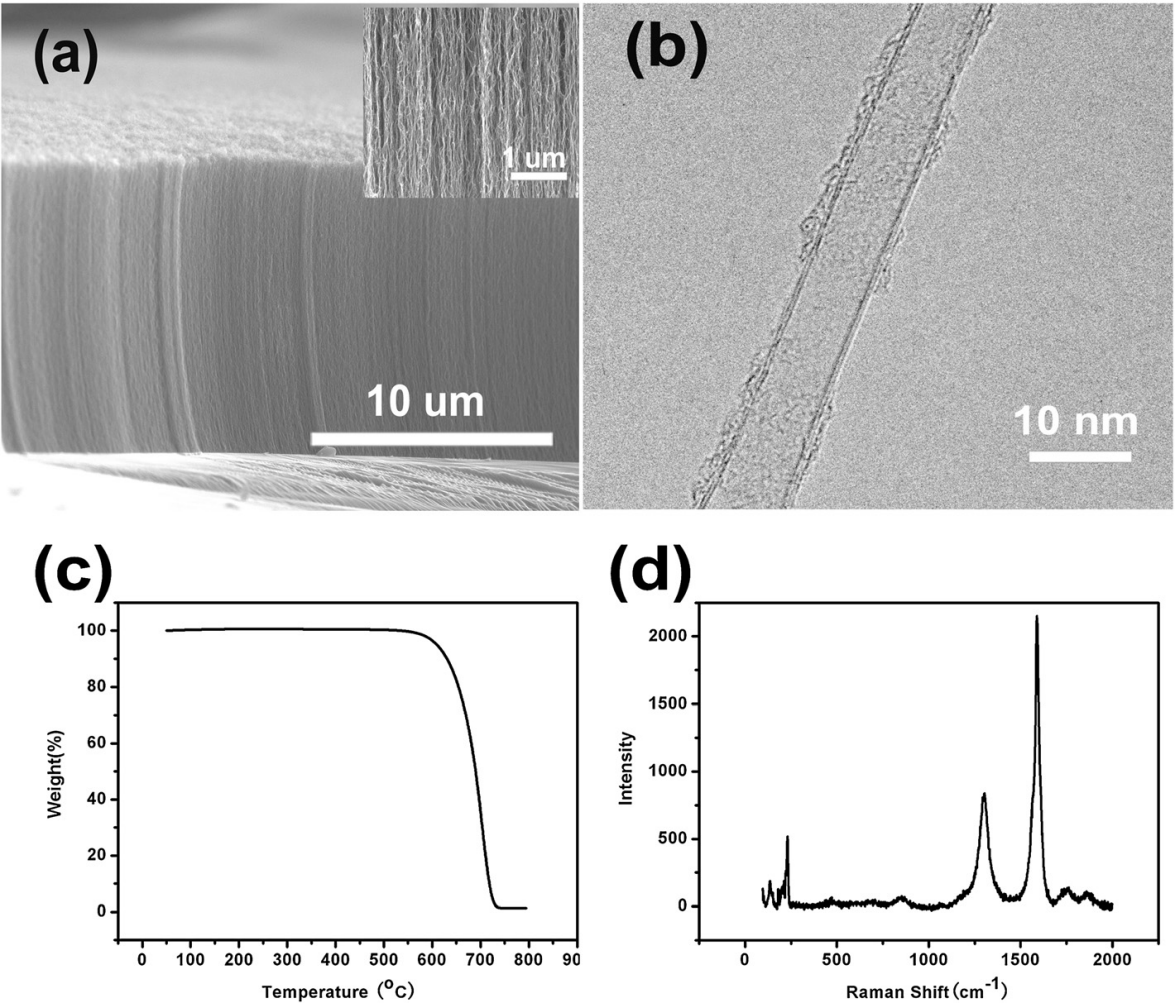
**Characterizations of vertically aligned CNTs. (a)** SEM image of the CNT forest. **(b)** HRTEM image of a typical CNT in the forests. **(c)** TGA analysis of the CNTs at a heating rate 5°C/min in air. **(d)** Raman spectra of the CNTs.

Figure 1a shows a scanning electron microscopy (SEM) image of a typical CNT forest grown by water-assisted CVD. The forest is about 10 μm in height, and the CNTs are highly aligned and continuous as shown in the inset of Figure 1a. Figure 1b presents a high-resolution transmission electron microscopy (HRTEM) image of a typical CNT in the forests. The diameter was around 7 nm, and the graphitic wall number was 3. Thermogravimetric analysis (TGA) at a heating rate of 5°C/min (Figure 1c) shows that there is no measurable residue in the sample heated over 750°C in air, suggesting a very high carbon purity of the CNTs. A G-band at 1,590 cm^-1^ and a D-band at 1,308 cm^-1^ in Raman spectra of the samples were shown in Figure 1d, which are caused by the in-plane vibration of graphite with an E_2g_ symmetry intra-layer mode and the defects in the nanotubes or amorphous carbon, respectively. The G-band to D-band intensity ratio of approximately 2.8 indicates a high crystallinity of the CNTs.

**Figure 2:**
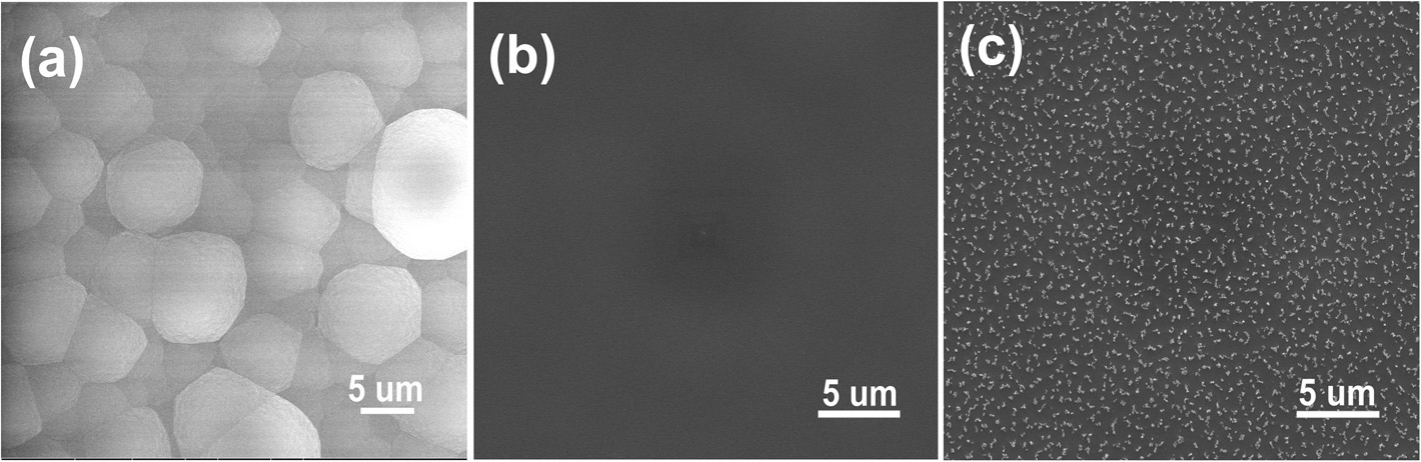
**SEM images of the VACNT/parylene composite membrane. (a)** SEM image of the top surface of VACNT/parylene composite membrane after parylene deposition. **(b)** SEM image of the top surface of VACNT/parylene composite membrane after annealing treatment (375°C for 1 h). **(c)** SEM image of the top surface of the VACNT/parylene composite membrane after Ar/O_2_ plasma etching.

**Figure 3:**
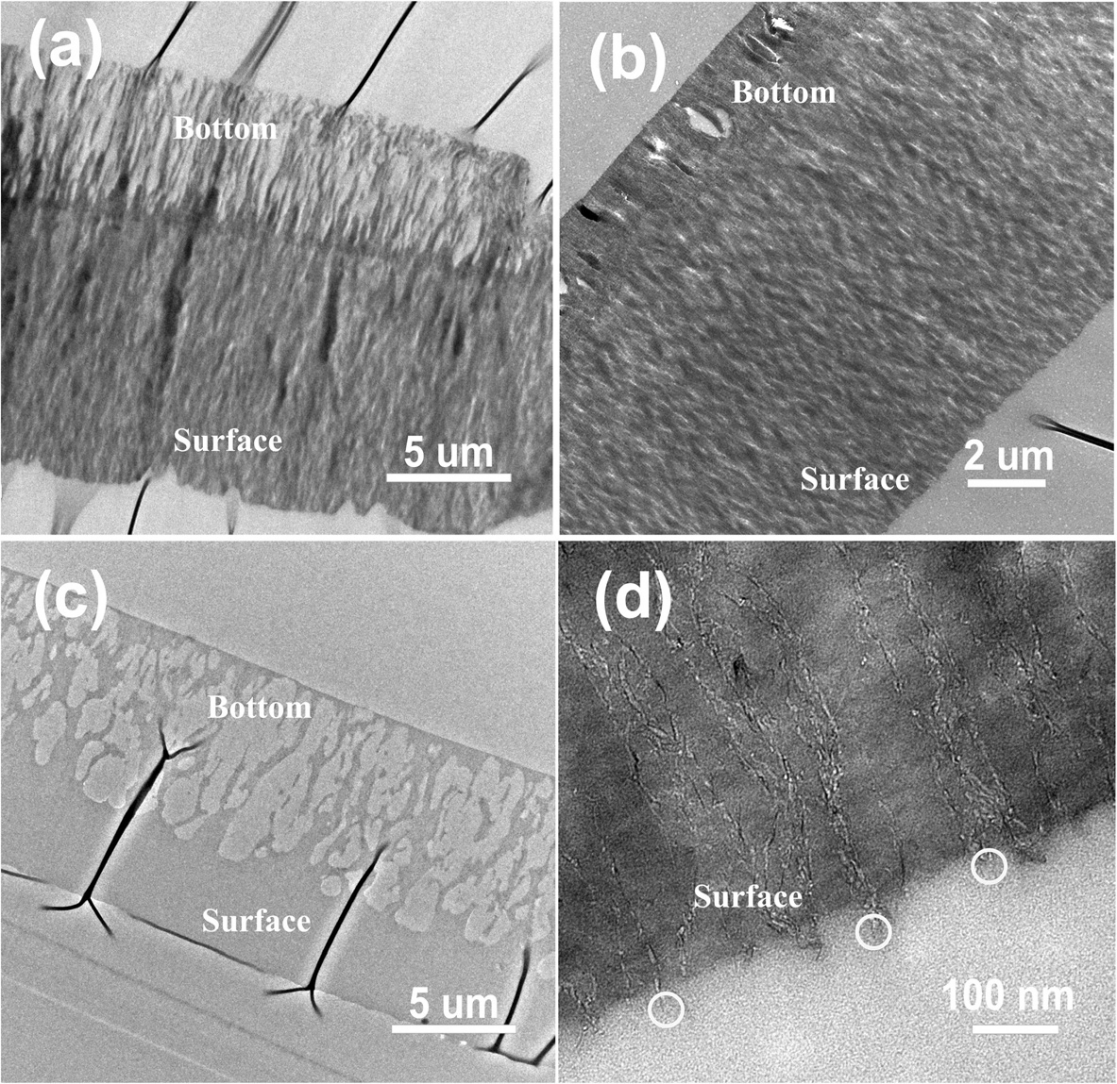
**TEM images of the VACNT/parylene composite membrane. (a-c)** Low-magnification cross-sectional TEM images of the VACNT/parylene composite membrane after annealing at 325°C, 375°C, and 425°C, respectively. **(d)** High-magnification cross-sectional TEM image of the VACNT/parylene composite membrane after annealing at 375°C for 1 h.

VACNTs were infiltrated with parylene by CVD [17]. Additional file 1: Figure S3 shows the schematic fabrication process of the VACNT/parylene composites. Specifically, the parylene monomers were transferred into the gaps among VACNTs in a vapor state and then polymerized *in situ* to form a gastight matrix of the membrane. Since there is no surface tension involved in this process, the vertical alignment of CNTs could be well maintained.Figure 2a shows SEM image of top surface of the as-prepared CNT/parylene composites. Clearly, the top surface of the membrane was covered with a continuous parylene coating. After parylene deposition, the VACNT/parylene composite samples were heat treated in Ar atmosphere to allow the parylene to reflow and to improve the impregnation of parylene. Three conditions were explored, and a relatively flat surface was observed after annealing at 375°C for 1 h, as shown in Figure 2b. Transmission electron microscopy (TEM) observation was carried out after embedding the VACNT/parylene sample in epoxy resin and slicing with ultramicrotome. CNT forests were found to be completely embedded in the polymer matrix, and no large voids were observed in the bulk of the composite after annealing at 375°C (Figure 3b). Treating at 325°C was not efficient to improve the infiltration of parylene, and a lot of voids were found in the section close to the bottom of VACNTs (Figure 3a). Figure 3c demonstrates TEM image of the composite after annealing at 425°C. Serious deformation of CNT forests and a lot of macroscopic defects were observed in the composite. These results indicate that annealing at an appropriate temperature was important for fabricating a composite membrane with the dense parylene matrix.

After annealing (at 375°C), the extra parylene covering tips of CNTs on the membrane surfaces was removed by Ar/O_2_ plasma-enhanced oxidation. Plasma etching was performed at 100 W for 90 min by using 71.4% O_2_ in the feed gas. Figure 2c shows SEM image of the top surface of VACNT/parylene composite after plasma etching. Large numbers of bright spots were found, which were believed to be the extending CNT tips agglomerated together, sine parylene was etched faster than CNTs by oxidative plasma [9-11]. HRTEM observation (Figure 3d) confirms the protruding of CNTs from the above of the composite surface after plasma treatment. Furthermore, the marked area highlighted the opened CNT tips, which provides a direct proof for the opening of the exposed CNTs by oxidative plasma. Subsequently, HF acid was used to remove the VACNT/parylene composite from the Si substrate to produce a freestanding membrane. Another 5-min plasma etching was performed on the backside to expose the CNTs from the bottom surface. After these procedures, freestanding composite membranes with vertically aligned CNTs embedded in the parylene matrix were successfully fabricated.

**Figure 4:**
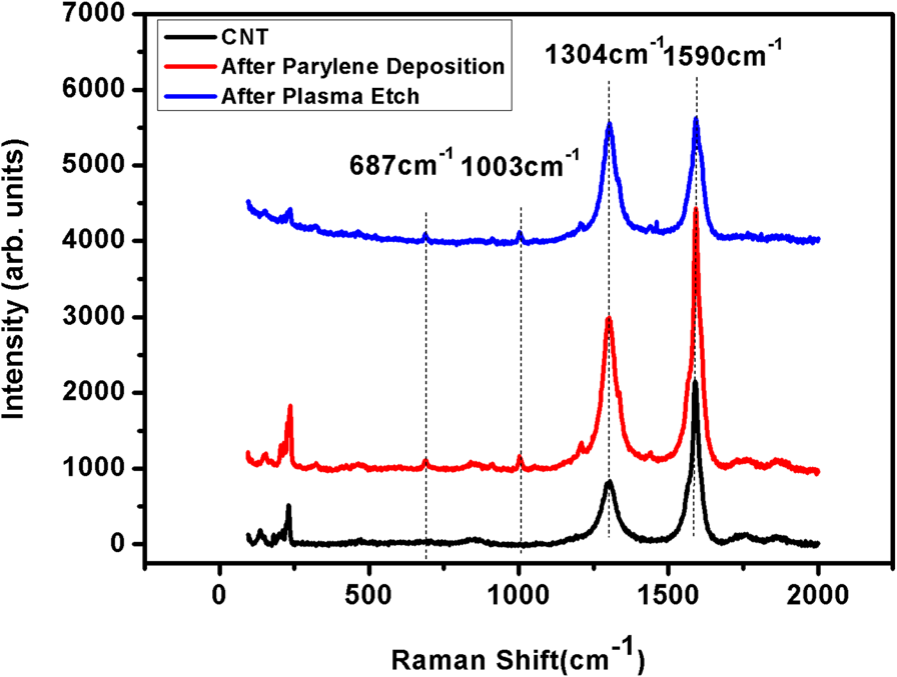
**Raman spectra of the CNTs and the composite membranes.** Raman spectra of the as-synthesized CNTs and VACNT/parylene (CP) composite membranes and composite membranes after plasma etching (PE).

Raman spectroscopy was employed to characterize the structure of CNTs during membrane fabrication. Figure 4 shows Raman spectra of the as-synthesized CNT forest, the VACNT/parylene composite membrane, and the composite membrane after plasma etching treatment. The G-band at 1,590 cm^-1^ is associated with the E_2g_ in-plane stretching vibration mode on the basal plane of graphite, which indicates the existence of crystalline graphitic carbon in the CNT samples. The peak at 1,304 cm^-1^ (D-band) is assigned to the imperfections in CNTs and amorphous carbon. The intensity ratio between G-band (*I*_G_) and D-band (*I*_D_) is sensitive to chemical modification and is a measure of the defects in CNTs. The *I*_G_/*I*_D_ ratio is determined to be 2.56 for the as-synthesized CNTs, suggesting good crystallinity of the CNT array grown by water-assisted CVD. As shown in Figure 4, the G-band and D-band peak positions do not change, and the two bands (1,003 and 687 cm^-1^) ascribed to parylene appear in the Raman spectrum of CNT array after parylene deposition. Although no distinctive change in terms of the Raman shift of G-band or D-band is found, the *I*_G_/*I*_D_ ratio decreases from 2.56 for the as-synthesized CNT to 1.02 for the composite membrane treated by plasma etching. The Raman analyses suggest that the deposition of parylene into the CNT array does not cause any damage to CNTs, while the plasma etching induced structural defects on CNT tips above the membrane surface.

**Figure 5:**
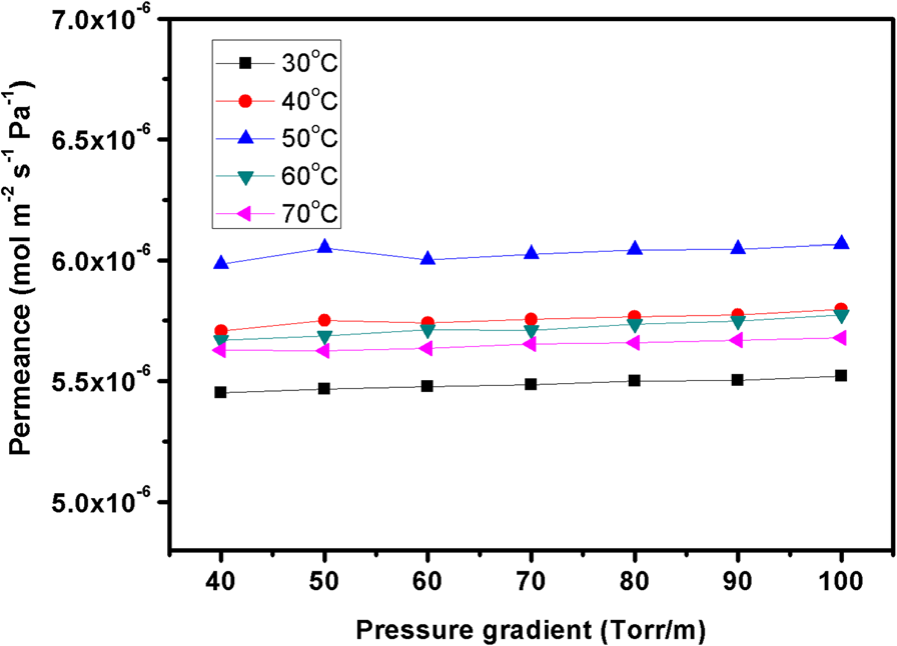
**Ar permeances through the membrane.** Argon permeances through VACNT/parylene membranes at different temperatures.

Figure 5 shows Ar permeances versus pressure gradient across the composite membrane at various temperatures. Obviously, at the temperature between 30°C to 70°C, the Ar permeance through the CNT membrane is independent of the applied pressure gradient. Permeances of other gases including H_2_, He, N_2_, O_2_, and CO_2_ show the same dependence on the feed pressure.

In general, gas transport through a porous membrane can be described by viscous flow, Knudsen diffusion, and surface diffusion [11,17,30,31]. Knudsen diffusion becomes prominent when the mean free path of the diffusing species is larger than the pore diameter. For most gases, the mean free path is significantly larger than the pore diameter of the CNT membrane (7 nm). Hence, one would expect the gas transport through the CNT membrane to be in the Knudsen regime [30,32]. The Knudsen permeance could be estimated using the following equation:

(1) $$P_{\mathrm{Kn}}=\frac{\Phi \epsilon_{p}}{\mathrm{\tau RTL}}{\left(\frac{8\mathrm{RT}}{\mathrm{\pi M}}\right)}^{0.5}\text{,}$$

where *P*_Kn_ is the Knudsen permeation (mol m^-2^ s^-1^ Pa^-1^), *ϵ*_p_ is the porosity, *τ* is the tortuosity, Φ is the inner diameter of CNT (m), *L* is the layer thickness (m), *M* is the molecular mass (kg mol^-1^) of the gas molecule, and *T* is the absolute temperature (K).The constant experimental permeances of the gases irrespective of the pressure gradient are consistent with the Knudsen model, which provide indirect but important evidence that the gas molecules do transport through the nanoscale interior channel of CNTs rather than the relatively large cracks in the membranes. This finding agrees well with the good impregnation of CNTs with the parylene, which has been demonstrated in Figure 3b.

**Figure 6:**
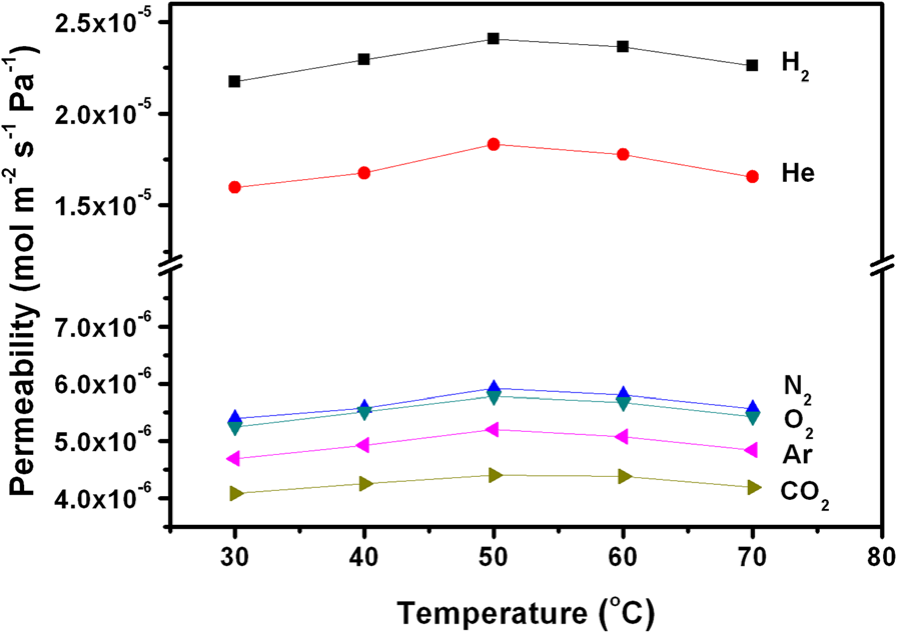
**Permeability of gases at different temperatures.** Temperature dependence of the gas permeances across the CNT composite membrane.

Temperature dependence of the gas permeances across the CNT composite membrane was explored, and the results were presented in Figure 6. According to the Knudsen theory (Equation 1), the gas permeance would decrease with increasing temperature. Surprisingly, our experimental permeances of all the gases firstly increased with raising the temperature up to 50°C and then decreased as the temperature further rose. Ge et al. also found similar dependence of gas permeance on the temperature in VACNT/epoxy membranes and attributed it to the contribution of both surface diffusion and Knudsen diffusion [11].

To investigate the enhancement of experimental permeances over theoretic prediction, the Knudsen permeances were computed using Equation 1. The parameters of the VACNT/parylene membranes are listed in Table 1 for calculating the Knudsen permeance. The membrane porosity *ϵ*_p_ ~ 0.0008 is estimated from the KCl diffusion experiments [30], as described in Additional file 1.

**Table 1:** Parameters of VACNT/parylene membranes

**Parameters**	**Values**
Thickness *I* (μm)	Approximately 10
CNT diameter *Φ* (nm)	Approximately 7
CNT tortuosity factor (*τ*)	Approximately 1
Areal porosity (*ϵ*_p_)	Approximately 0.0008

**Figure 7:**
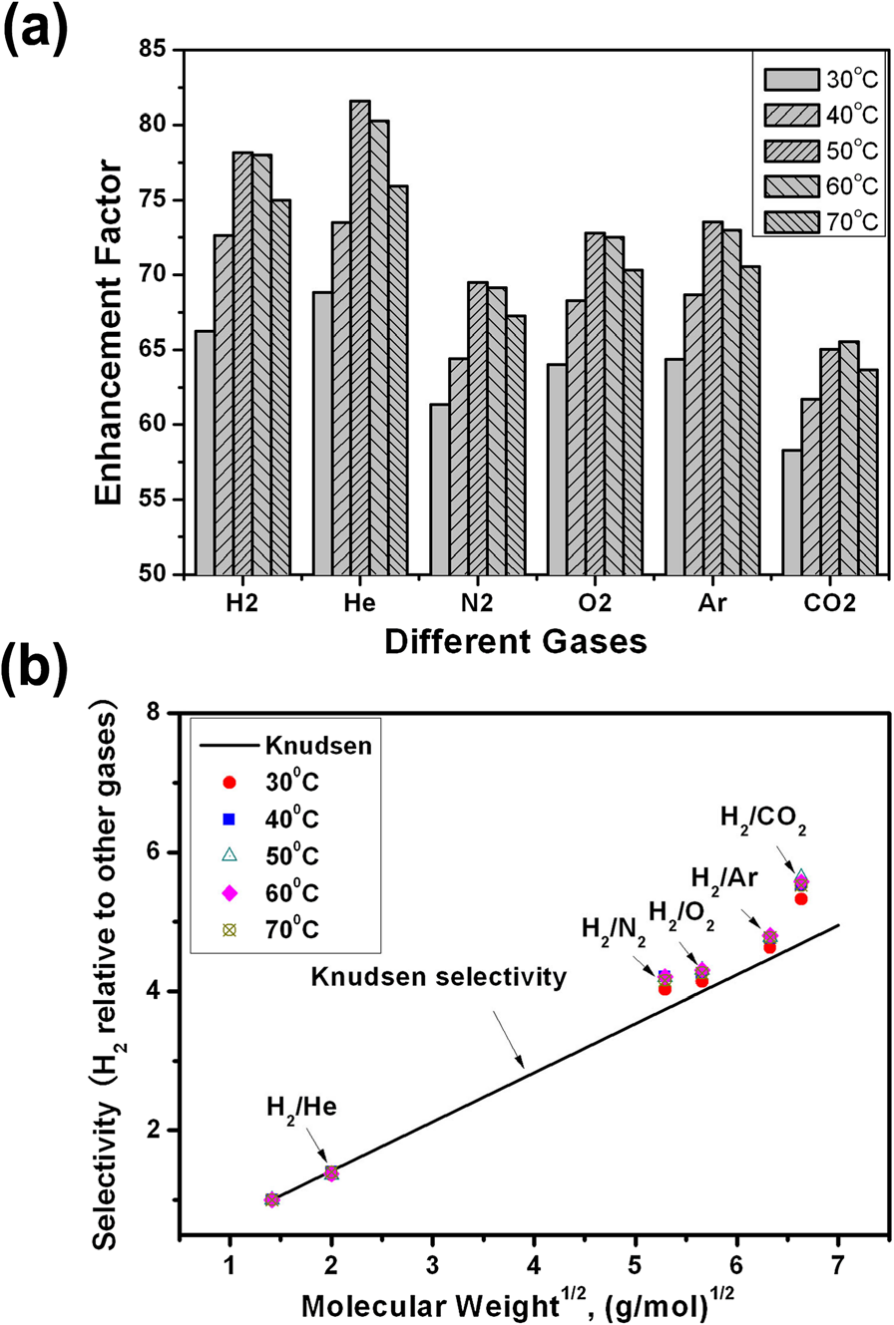
**Enhancement factors and the selectivity. (a)** Enhancement factors of gases under different temperatures. **(b)** The selectivity of hydrogen to gases.

The permeance enhancement factor is defined as the ratio of experimental permeance to the Knudsen permeance. For the six gases, the enhancement factors through VACNT/parylene membranes were over 60 under different temperatures (Figure 7a), suggesting much higher transport rate than the Knudsen diffusion. This observation is consistent with the experimental results of VACNT composite membranes reported previously, where enhancement of 1 to 2 order of magnitude over the Knudsen permeance was found [9-12]. Such significant enhancement in gas diffusion is attributed to the smooth VACNT channels in the membranes where backscattering molecular collisions do not occur. The forward momentum of gas flow is unchanged upon gas transport in the CNT channels. The skating-like gas transport along the VACNT channels is much different with the randomly scattered Knudsen diffusion, resulting in very high flow velocity. The specular feature of momentum transfer results in the significant increases of gas diffusivities which are even much higher than those predicted by the kinetic theory [30].

Interestingly, the enhancement factors of each gas show a similar dependence on temperature with the permeance. For most gases, the enhancement factor firstly increased as the temperature rose up to 50°C and then decreased with further increasing temperature. The changed enhancement factor with temperature and the temperature-dependent gas permeance both suggested that the gas diffusion in CNT channels does not fully conform to the Knudsen diffusion kinetics, and other diffusion mechanisms of gas molecules might exist. It is well established that the surface-adsorption-based diffusion in microporous membranes is an activation process, following the Arrhenius-type equation [33,34]. Therefore, the increased permeance and enhancement factor with the temperature below 50°C indicated that surface diffusion might also play an important role in the total gas diffusion through our CNT/parylene membranes. Since the surface diffusion is thermally activated, its contribution to the total diffusivity was expected to rise with increasing temperature, which could lead to the increase in gas permeance and enhancement factor. However, when the temperature was over 50°C, gas adsorption on the CNT walls was attenuated and thus the contribution of surface diffusion to overall permeance decreased gradually with the temperature increment. Accordingly, the gas permeance and the enhancement factor over Knudsen kinetics decreased with further increasing temperature.

Figure 7b shows selectivity of hydrogen relative to other gases (He, Ar, N_2_, O_2_, CO_2_). Based on Knudsen diffusion, the gas selectivity is inversely proportional to the square root of the molecular weight ratio. For different gas pairs, the selectivity values are scattered around the Knudsen selectivity regime. However, the different selectivities at various temperatures demonstrated a derivation from the Knudsen kinetics, indicating the presence of other diffusion mechanisms beside Knudsen, such as surface diffusion.

## Conclusions

In summary, an effective method to prepare flexible and robust VACNT/parylene composite membranes has been successfully developed by infiltrating CNT forests with parylene and exposing CNT tips through plasma etching. Transport properties of six gases across the composite membrane were explored, and gas permeances were found to be over 60 times higher than the Knudsen model prediction, which was attributed to the atomically smooth inner walls of CNTs. Investigation on temperature dependence of the gas permeances showed a tendency of first increase and subsequent decrease, and the permeance peaks around 50°C. H_2_ selectivity relative to other gases was around the Knudsen regime but also dependent on temperature. Discrepancy in the temperature dependences of the gas permeance and the selectivity with the Knudsen model indicates the existence of non-Knudsen transport and thermally activated surface diffusion. Further modeling and experimental investigations are still necessary to elucidate the non-Knudsen diffusion in the CNT composite membranes.

## Competing interests

The authors declare that they have no competing interests.

## Authors’ contributions

LZ carried out the growth of the samples and analysis of the results and drafted the manuscript. BZ and JY conceived the study, participated in its design and coordination, and helped to draft the manuscript. XW and GZ helped to draft the manuscript. All authors read and approved the final manuscript.

## Authors’ information

LZ is a carbon research scientist and a postgraduate of the University of Shanghai for Science and Technology. JY is a carbon research scientist and the head of the Advanced Carbon Materials Team at the University of Shanghai for Science and Technology.

## Supplementary Material

Additional file 1 **KCl diffusion experiments for porosity estimation. Figure S1**. The relation between the conductivity of solution and the KCl concentration. **Figure S2**. The conductivity of the permeate solution as a function of time. **Figure S3**. Schematic of the preparation of VACNT/parylene membrane. Click here for file
