# Supplementary material for: Temperature-dependent gas transport performance of vertically aligned carbon nanotube/parylene composite membranes
Source: Nanoscale Res Lett. 2014 Aug 28;9(1):448. doi: 10.1186/1556-276X-9-448 (PMC4158770; doi:10.1186/1556-276X-9-448)
Supplement: Additional file 1 — KCl diffusion experiments for porosity estimation. Figure S1. The relation between the conductivity of solution and the KCl concentration. Figure S2. The conductivity of the permeate solution as a function of time. Figure S3. Schematic of the preparation of VACNT/parylene membrane. [file 1556-276X-9-448-S1.doc]

Supplementary Information

**Temperature-dependent gas transport performance of vertically-aligned carbon nanotube/parylene composite membranes**

Lei Zhang ^1^, Junhe Yang ^1^,* , Xianying Wang ^1^, Bin Zhao ^1^,*, Guangping Zheng ^2^

^1^ *School of Materials Science and Engineering, University of Shanghai for Science and Technology, Shanghai 200093, China*

^2^ *Department of Mechanical Engineering, The Hong Kong Polytechnic University, Hung Hom, Kowloon, Hong Kong, China*

KCl Diffusion Experiments for Porosity Estimation

Fig. S1 shows the relation between the conductivity of solution and the KCl concentration. The conductivity of the permeate solution as a function of time is shown in Fig. S2.


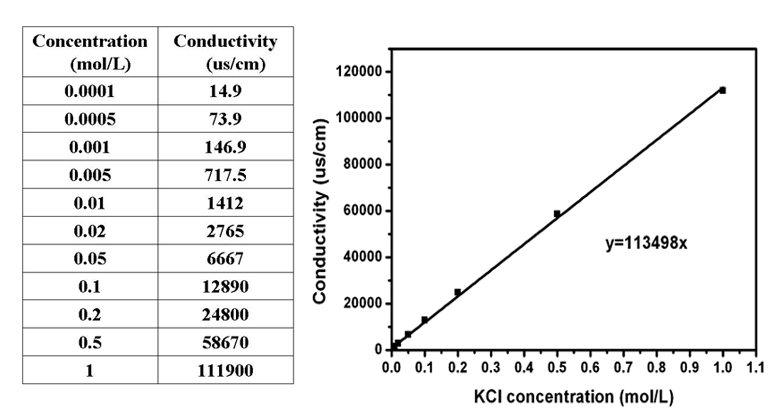


**Fig. S1.** The relation between the conductivity of solution and the KCl concentration.


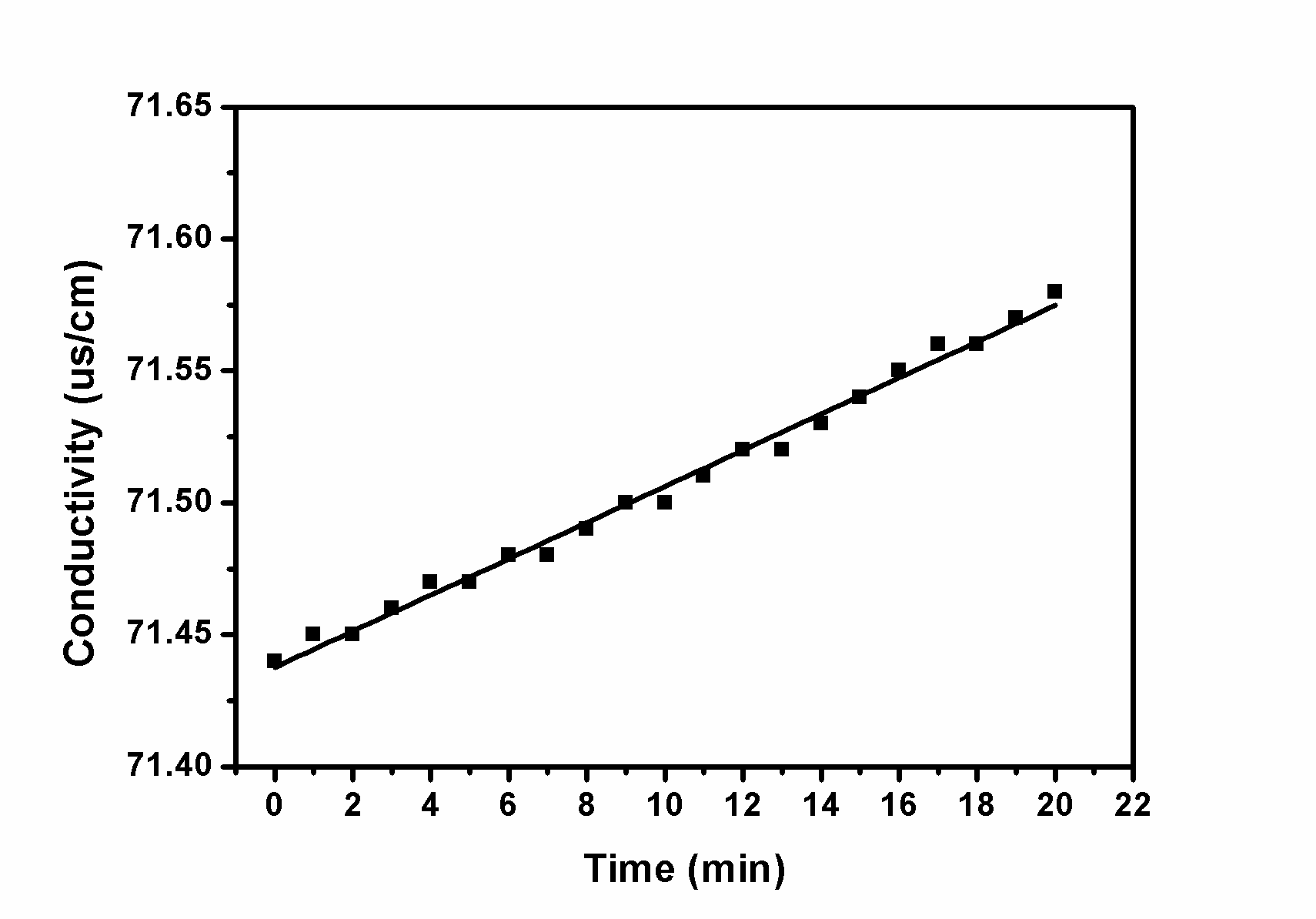


**Fig. S2.** The conductivity of the permeate solution as a function of time.

The conductivity data is converted into flux data using a calibration relation between electrical conductivity and KCl concentration (in ppm), as follows:

$\text{J}=\frac{(\text{K}_{1}-\text{K}_{0})}{113498\times\text{S}}\times\text{V}$ , (1)

where J is the experimental steady-state flux of KCl (mol/s), K_1_=71.59 us/cm is the final conductivity, K_0_=71.43 us/cm is the starting conductivity, S=1200 s is the testing time, V=8.67x10^-3^ L is the volume of solution. Therefore we have $\text{J}=1.02\times10^{-11}mol/s$.

The porosity (*ε*_p_) of the membrane can be calculated by the following equation

$\varepsilon_{\text{p}}=\frac{\left(\frac{J\times I}{D\times C}\right)}{A_{m}}$, (2)

where J is the experimental steady-state flux of KCl (1.02x10^-11^ mol/s), I is the membrane thickness (10x10^-6^ m), D is the bulk diffusivity of KCl at 25 ^o^C (~1.87x10^-9^ m^2^/s), C is the concentration of the feed solution (1.0x10^-2^ mol/L) and A_m_~7.06x10^-6^ m^2^ is the membrane area exposed in the diffusion experiment.

Fig. S3 shows the schematic of preparation of the VACNT/parylene membrane.

In the deposition of parylene, the large parylene molecules were first cracked into relatively small molecules at high temperature and were then transferred into the reactor chamber where the monomers polymerized to form parylene polymer at low temperature.

The CNTs retain highly aligned structures and remain continuous from the bottom to the top surfaces in the resulting composite membrane.


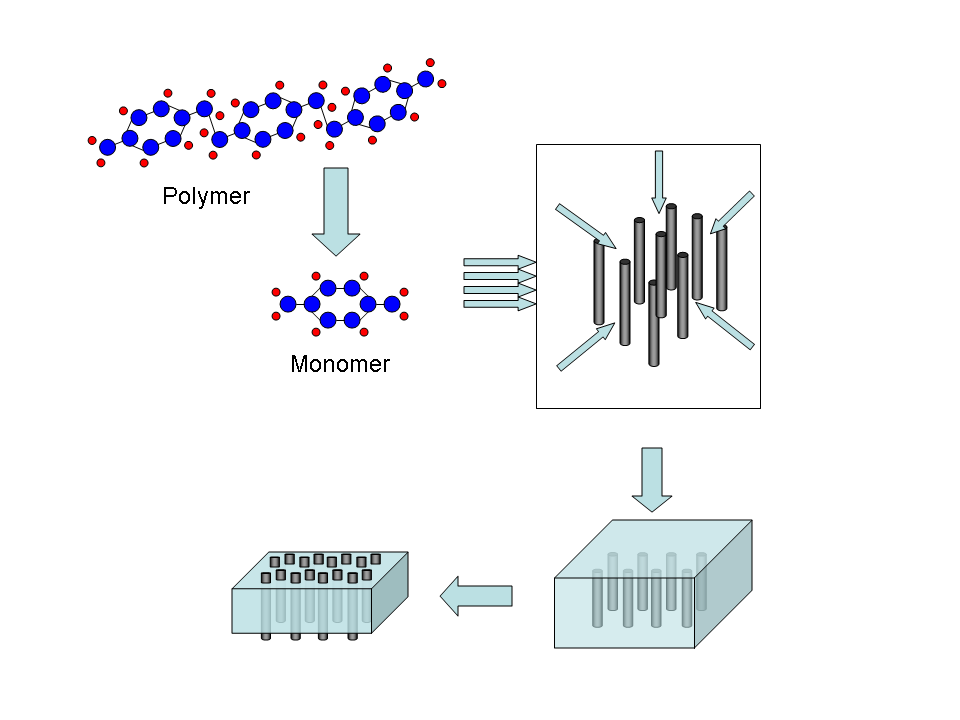


**Fig. S3.** Schematic of the preparation of VACNT/parylene membrane.
